# An E-Health Solution for People With Alcohol Problems

**Published:** 2011

**Authors:** David H. Gustafson, Michael G. Boyle, Bret R. Shaw, Andrew Isham, Fiona McTavish, Stephanie Richards, Christopher Schubert, Michael Levy, Kim Johnson

**Keywords:** Alcohol use disorders (AUDs), treatment method, self-management, continuing care, information and communication technologies (ICTs), Alcohol Comprehensive Health Enhancement Support System (A-CHESS) Program, telecommunication technology, smartphone, literature review

## Abstract

Self-management of chronic diseases has been a research focus for years. Information and communication technologies (ICTs) have played a significant role in aiding patients and their families with that management task. The recent dramatic increase in smartphone capabilities has expanded the potential of these technologies by facilitating the integration of features specific to cell phones with advanced capabilities that extend the reach of what type of information can be assessed and which services can be provided. A recent review of the literature covering the use of ICTs in managing chronic diseases, including addiction, has examined the effectiveness of ICTs, with an emphasis on technologies tested in randomized controlled trials. One example of an addiction-relapse prevention system currently being tested is the Alcohol Comprehensive Health Enhancement Support System (A-CHESS) Program.

Approximately 17 million people in the United States suffer from alcohol use disorders (AUDs) (i.e., alcohol abuse and/or dependence), yet only 10 percent of them receive treatment (Substance Abuse and Mental Health Services Administration 2009). Decreased funding and high staff turnover resulting from low wages and poor working conditions exacerbate the problem. One characteristic of AUDs and other addictive behaviors is their chronically relapsing nature. Relapse reduces quality of life, puts great strains on family relationships, and burdens society through crime, health care costs, and reduced productivity (Ettner 2006; Slaymaker and Owen 2006). Although continuing-care approaches may reduce the risk of relapse, experience suggests that widespread use of these approaches is limited. For example, costs, geographic distance, and lack of time reduce patient participation in such programs. And even if patients participate, the programs frequently simply mirror the treatment approaches provided in the initial intensive care rather than offer a tailored continuing-care approach specifically addressing problems that patients may experience during early recovery in community settings. Improvements to the existing system may help, and solutions currently being considered (e.g., integration with primary care) potentially can improve treatment effectiveness and expand its reach. However, it is unlikely that these strategies can address the full scope of the problem. Similarly, an approach called Recovery Oriented Systems of Care (Halvorson et al. 2009) offers important guidelines for improvement (including self-management and family involvement) but requires dramatic transitions in thinking and practice that may take years to implement.

Chronic disease self-management has been a research focus in different medical fields for years. A small-group, self-management program developed by Lorig and colleagues (2004) that focuses on skills mastery provided by peer facilitators, group persuasion, and symptom reinterpretation is a leading evidence-based example. The program’s face-to-face version significantly improves health behaviors and health status and reduces health care use (Lorig et al. 2004). Conversely, a randomized controlled trial (RCT) of an Internet-based version of this program found improvement in some quality-of-life measures but not in health care use (Lorig et al. 2006).

In the substance abuse field, McKay’s (2009) review of the literature regarding continuing care for substance use disorders found that two features were associated with effective interventions (McKay 2009; for more information see the article by McKay and Hiller-Sturmhöfel, pp. 356–370 in this issue):

- Long duration of care (i.e., at least 12 months).
- Efforts to actively intervene to change the patient’s behaviors, such as involving a spouse or partner; delivering services in the patient’s home as a complement to face-to-face care; telephone delivery of the intervention; proactively looking for patients to ensure they stay in care and to get them back into treatment if needed; and linking patients to continuing-care services through case management and outreach.

In addition, McKay and colleagues (2004) conducted an RCT to evaluate a 12-week, telephone-based, continuing-care intervention (15–30 minutes in length) for people dependent on alcohol and/or cocaine who had completed intensive outpatient programs. The investigators monitored the participants’ substance use status and progress toward selected goals via telephone calls, identified high-risk situations, and developed and rehearsed coping behaviors. In addition, participants had access to a weekly support group for 4 weeks. At 24 months, the intervention resulted in higher self-reported abstinence, fewer heavy drinking days, and lower liver enzyme values in alcohol-dependent participants than did a comparison treatment of cognitive–behavioral therapy/relapse prevention. Finally, a subsequent 18-month telephone study by McKay (in press) found that for people who had participated in intensive outpatient treatment, adding a counseling component to treatment monitoring and feedback produced better results for any alcohol use and heavy drinking days.

This article will look at some new approaches to continuing care for patients with AUDs and other addictions, particularly those interventions that rely on newer technologies (i.e., information and communication technologies [ICTs]). This discussion also will present in more detail an approach called the Comprehensive Health Enhancement Support System (CHESS) that was developed at the University of Wisconsin’s Center for Health Enhancement Systems Studies. In addition, the results of a literature review of studies covering the use of ICTs in managing chronic diseases, including addictions, are summarized.

## Use of New Technologies in the Long-Term Treatment of Patients With AUDs

As the work described in the preceding paragraphs suggests, ICT-based approaches to support self-management may be able to help in the long-term treatment of patients with AUDs and other addictions. Other studies support this assumption. For example, research has demonstrated that people suffering from addictions view computer-based interventions as helpful in managing recovery (Cunningham et al. 1999). Moreover, patients typically acknowledge more drug use and psychiatric symptoms online than through face-to-face interviews (Rosen et al. 2000). Computer-based brief interventions also have been shown to increase motivation and reduce problem drinking (Hester et al. 2005; Murray et al. 2007). Simpson and colleagues (2005) demonstrated that interactive voice response (IVR) and other monitoring mechanisms can be used to collect data from people with alcohol-related disorders and that these data then can initiate support to prevent relapse. For example, reminder systems and alerts can alert a patient, family member, and/or clinician so they can take steps to prevent or deal with a potentially significant problem (DuBenske et al., in press). Similarly, bulletin boards and e-mail tools can be powerful sources of social support (Han et al. 2008, 2009, 2010 in press; Kim et al. 2010; Shaw et al. 2007, 2008*a*,*b*).

A new development that may come to play a role in long-term management of chronic diseases, including drug use disorders, are smartphones—mobile phones offering advanced capabilities, often with computer-like functionality. Results from studies using smartphones are only just beginning to become available, but earlier experiences with regular cellular phones already provide some insights into their potential. For example, these older studies have demonstrated that compliance with the interventions can be high. In one study (Searles et al. 2002), alcohol-dependent participants responded to over 93 percent of calls made. Although this finding is encouraging, it is important to recognize that alcohol abuse often is associated with marked deficits in cognitive functioning (Sullivan et al. 2000), including reduced ability to read (Beatty et al. 1996). Hence, intervention effects found with other chronic diseases may not hold for AUDs. Given the potential benefit and many questions surrounding the use of this technology for AUD treatment, more research on smartphone-based interventions is needed. A search of the National Institutes of Health (NIH) Research Portfolio Online Reporting Tools (REPORT) database of funded NIH grants identified 12 randomized trials involving smartphones currently under way; of these, 4 focused on weight and activity; 2 on substance use disorders; 2 on AIDS/HIV; 2 on mental health; and 1 each on vaccinations, smoking, and drugs. One of these studies involves the Alcohol–Comprehensive Health Enhancement Support System (A-CHESS), a smartphone-based relapse-prevention system developed by the authors of this article. The following section describes the CHESS approach in more detail and the sidebar (p. 329) summarizes the services provided by the A-CHESS.

### CHESS

The recent, dramatic increase in smartphone capabilities has expanded the potential of ICTs in the management of chronic diseases by facilitating the integration of technological features specific to cell phones with advanced capabilities such as global positioning systems (GPS), text messaging, and cameras, extending the reach of what can be assessed and provided. The CHESS is one example of an approach using smartphone versions for a variety of applications, including support of inner-city teenagers with asthma (Wise et al. 2010), colon cancer survivorship, and relapse prevention in alcoholism (Gustafson et al., in press).

The CHESS has been developed at the Center for Health Enhancement Systems Studies at the University of Wisconsin–Madison, which has developed and tested ICTs to help people cope with a range of serious health issues. As one of five National Cancer Institute (NCI)-designated Centers of Excellence in Cancer Communication Research, investigators at the Center focus on researching and developing innovative health systems that optimize individuals’ health behaviors, quality of life, and access to health services. CHESS is the Center’s main developmental platform; it is an umbrella name for several computer-based e-health systems that each have a different focus (e.g., breast cancer, caregivers of children with asthma, or HIV) (available at: www.chess.wisc.edu). CHESS programs are constructed to meet user needs that are identified in studies of the target population (Gustafson 2004). They provide information, adherence strategies, decision-making tools, reminders, monitoring with alerts, and social support services in attractive, easy-to-use formats and are designed to eliminate the need for complicated Internet searches. In recent smartphone versions, most content is presented in both audio and text formats to enhance access for people with literacy problems.

**Description of A-CHESS Services**

The Alcohol–Comprehensive Health Enhancement Support System (A-CHESS) is designed to be compatible with two models of how people can change their behaviors—the self-determination theory (Larimer et al. 1999) and a model developed by Witkiewitz and Marlatt (2004) that describes stages preceding relapse and stage-appropriate change methods to prevent relapse. The figure illustrates how the A-CHESS follows these two models and illustrates the services the program offers. These services encompass a wide range of components, as described in the following sections.

**Setup**

Before discharge from residential care, patients assigned to A-CHESS are equipped with a smartphone (EVO) containing A-CHESS content. The counselor enters setup information to tailor A-CHESS operation. This information includes the following:

- Patient demographics;
- The patient’s level of self-efficacy and coping style (Steptoe 1989);
- Healthy events of interest to the patient;
- Therapeutic goals and care plan;
- High-risk locations that have been problematic to the patient;
- Benefits the patient gets from using alcohol, reasons they fear relapse, and poignant memories from previous use; and
- Key triggers and interventions likely to help deal with those triggers.

With the patient’s agreement, the setup establishes protocols for contact in different scenarios, such as when check-in (monitoring) is scheduled, the “panic button” is pushed, an appointment or medication reminder occurs, and when the phone’s global positioning system (GPS) detects the patient approaching a high-risk location.

**Contacts**

The A-CHESS system allows for two types of contacts—emergency and nonemergency contacts. The emergency contact is triggered by the panic button, which when pushed initiates support to prevent a relapse. Patients needing immediate help can press this button to reach counselors or get help from A-CHESS. The GPS location tracker also can trigger the panic button if the patient approaches a trigger location. Nonemergency contact can occur in three ways:

- Patients can use A-CHESS when they wish.
- After 10 days of inactivity, A-CHESS will send a message to the patient and care manager to encourage A-CHESS use.
- Each week, A-CHESS conducts a “check in” by displaying a brief survey on the phone’s screen (with audio overlay). This survey serves to obtain patient data on recent alcohol and other drug use, status on five protective factors and five risk factors taken from the Brief Alcohol Monitor (BAM) (Marlatt and George 1984), and desire to re-enter treatment. A-CHESS uses the check-in information for triage and feedback (see below). The patient’s care manager receives a summary report of the check-in data whenever they wish, on the day before a scheduled appointment, and whenever a patient reports a lapse or desire to re-enter treatment.

**Triage and Feedback**

Triage and feedback are intended to derail the relapse process by providing people with just-in-time, tailored information about recovery coping skills (Kreuter and Wray 2003; Strecher et al. 1994). Using data collected during setup and check-in, A-CHESS provides optional links to relevant A-CHESS resources. For patients who experience problems managing BAM protective or risk factors, A-CHESS reminds them of skills to use. It offers relaxation exercises, connections to online peer support, and links to a healthy-event newsletter; starts a diversionary activity; and contacts a counselor.

**Social Support**

Social support is essential in the management of any chronic disease and also is an integral part of A-CHESS. The goal is to cultivate a support network to help the patient develop positive addictions, substitute indulgences, and find support during a lapse (Stalcup et al. 2006; Walton et al. 2003). A-CHESS can provide social support through several means:

- *Discussion groups.* Patients can exchange emotional support and information with others assigned to their A-CHESS study arm via online bulletin board or text messaging (Alemi et al. 1996; Ouimette et al. 2001, 2003). Guidelines for appropriate use of discussion groups are stressed in training. Discussions are monitored to identify and act on inappropriate usage.
- *Ask an expert.* Patients who request information and advice receive a response within 24 hours (weekdays) from addiction experts. As with discussion groups, responses of general interest are rendered anonymous and provided for all to view.
- *Personal stories* (written and video interviews) by patients and families address strategies to overcome barriers to addiction management.
- *Mobile social software* allows users to text their location to physically nearby, preapproved friends, family, and peers so that they can respond to a request for help.

**Information Services**

A-CHESS uses check-in data to provide competence-building resources on a just-in-time basis or at a time of the patient’s choosing (e.g., when the patient experiences warning signals of relapse, needs to increase lifestyle balance, or requires stimulus control techniques to curb cravings). These resources include the following:

- *Instant Library.* Because full-length articles may be hard to read on a smart phone, A-CHESS provides audio summaries of key articles and chapters and manuals on addiction management.
- *A Medication* section provides information about addiction pharmacotherapies, ways to reduce side effects, and other barriers to adherence.
- *Questions & Answers* offers brief answers to hundreds of questions about addiction, with links to other A-CHESS services that provide more detail.
- *Web Links* allow patients to access recommended addiction-related Web sites, with information on the sites’ strengths and weaknesses.

**Figure:**
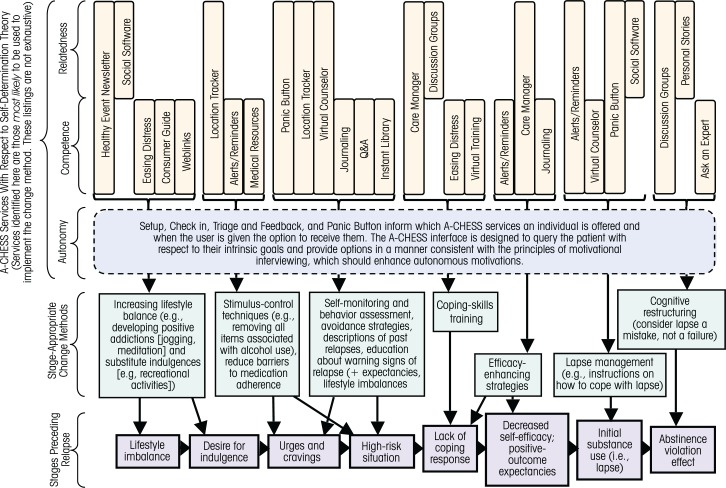

**Additional Tools**

Several additional tools are available with the A-CHESS system to reduce the risk of relapse and support the patient’s long-term disease management. These include the following:

- *Easing Distress* includes relaxation exercises.
- *Location Tracking* uses the smart-phone’s GPS to initiate rescue when the patient approaches a high-risk location. GPS also locates and provides maps to nearby meetings (e.g., of Alcoholics Anonymous) and treatment providers in emergency situations.
- *Reminders* provide timely text and audio reminders of medications, significant milestones, reasons for quitting, and inspirational messages.
- *Healthy Event Newsletter* populates the patient’s calendar with recent news and healthy activities that he or she expressed interest in during setup and links the patient to peers who share similar interests (Meyers et al. 2003).
- *Care Management* calls are scheduled with the patient’s care manager (Godley et al. 2002; McLellan et al. 1999; Rapp et al. 1992). Before each call, A-CHESS (with the patient’s permission) e-mails the counselor time graphs of the patient’s check-in data. The care manager reviews these reports, provides tailored education, and creates tailored links to relevant A-CHESS material. The patient and care manager can e-mail or text each other via a pre-programmed button. Additionally, the care manager receives notification if the patient’s check-in data exceed a threshold on key indicators or if the patient indicates that he or she needs to resume treatment (Sullivan et al. 1992).

ReferencesAlemiFStephensRCJavalghiRGA randomized trial of a telecommunications network for pregnant women who use cocaineMedical Care3410 Suppl.OS10OS201996884393310.1097/00005650-199610003-00002GodleyMDGodleySHDennisMLPreliminary outcomes from the assertive continuing care experiment for adolescents discharged from residential treatmentJournal of Substance Abuse Treatment231213220021212746510.1016/s0740-5472(02)00230-1GustafsonDHMcTavishFHawkinsRComputer support for elderly women with breast cancerJAMA: Journal of the American Medical Association2801513051998979430010.1001/jama.280.15.1305KreuterMWWrayRJTailored and targeted health communication: Strategies for enhancing information relevanceAmerican Journal of Health Behavior27Suppl 3S227S23220031467238310.5993/ajhb.27.1.s3.6LarimerMEPalmerRSMarlattGARelapse prevention. An overview of Marlatt’s cognitive-behavioral modelAlcohol Research & Health232151160199910890810PMC6760427MarlattGAGeorgeWHRelapse prevention: Introduction and overview of the modelBritish Journal of Addiction7942612731984659502010.1111/j.1360-0443.1984.tb00274.xMcLellanATLewisDCO’BrienCPKleberHDDrug dependence, a chronic medical illness: Implications for treatment, insurance, and outcomes evaluationJAMA: Journal of the American Medical Association284131689169520001101580010.1001/jama.284.13.1689MeyersRJSmithJELashDNThe Community Reinforcement ApproachRecent Developments in Alcoholism16183195200312638638OuimettePHumphreysKMoosRHSelf-help group participation among substance use disorder patients with posttraumatic stress disorderJournal of Substance Abuse Treatment201253220011123972510.1016/s0740-5472(00)00150-1OuimettePMoosRHFinneyJWPTSD treatment and 5-year remission among patients with substance use and posttraumatic stress disordersJournal of Consulting and Clinical Psychology71241041420031269903610.1037/0022-006x.71.2.410RappRCSiegalHAFisherJHA strengths-based model of case management/advocacy: Adapting a mental health model to practice work with persons who have substance abuse problemsNIDA Research Monograph127799119921436007StalcupSAChristianDStalcupJA treatment model for craving identification and managementJournal of Psychoactive Drugs38218920220061690345810.1080/02791072.2006.10399843SteptoeAAn abbreviated version of the Miller Behavioral Style ScaleBritish Journal of Clinical Psychology28Pt 21831841989274305810.1111/j.2044-8260.1989.tb00830.xStrecherVJKreuterMDen BoerDJThe effects of computer-tailored smoking cessation messages in family practice settingsJournal of Family Practice39326227019948077905SullivanWPWolkJLHartmannDJCase management in alcohol and drug treatment: Improving client outcomesFamilies in Society7341952031992WitkiewitzKMarlattGARelapse prevention for alcohol and drug problems: That was Zen, this is TaoAmerican Psychologist59422423520041514926310.1037/0003-066X.59.4.224

Several randomized trials found that CHESS approaches significantly improved quality of life (e.g., Gustafson et al. 2001). For instance, CHESS-based disease management programs were more effective than open access to Internet for improving outcomes of breast cancer patients (Gustafson et al. 2008) as well as improved quality of life and reduced costs for HIV-infected patients (Gustafson et al. 1999). In population studies, CHESS was a highly popular and low-cost source of support for underserved breast cancer patients (Gustafson et al. 2005) as well as elderly patients (Gustafson et al. 1998).

A-CHESS is a smartphone-based relapse prevention system designed to address three key constructs that, according to self-determination theory (Deci and Ryan 2002), are essential for lasting change, including coping competence, social support, and autonomous motivation. By being based on smartphones rather than personal computers, A-CHESS offers widespread access and focuses on alcohol-dependent patients leaving residential care. It has an optional audio delivery and is customizable to proffer services tailored to prevent relapse. The program enhances coping competence through timely monitoring to assess/communicate risk of relapse, alerts to encourage adherence to therapeutic goals, and individualized addiction-related materials and tools that focus on the needs of the particular patient. In addition, it offers GPS services and location-based resources to initiate rescue services if the patient is nearing a high-risk location. Social support is offered through communication with peer support groups and addiction experts, as well as a one-touch communication to connect with a care manager. Moreover, the system communicates the information in a respectful manner and offers resource options that patients can select when needed, thereby enhancing the patients’ autonomy.

A-CHESS is one of three CHESS smartphone systems currently being evaluated in RCTs. It is being tested in an RCT funded by the National Institute on Alcohol Abuse and Alcoholism (NIAAA) in two treatment centers as well as in pilot tests with a drug court and families of returning veterans dealing with alcohol abuse. The A-CHESS RCT evaluates the intervention’s long-term impact on risky drinking days, cravings, negative affect, negative consequences, and days of abstinence. Mediation analyses^1^ will examine the mechanisms of effect, whereas moderation analyses will examine the differential effect of A-CHESS in patients of different genders and level of social support.

## Literature Review of ICT-Based Interventions in Chronic Diseases

With growing acceptance of AUDs and other addictions as chronic diseases (McLellan et al 2000), it is useful to examine the effectiveness of ICT-based interventions—particularly those involving smartphones because of the growing potential of that technology in supporting recovery—in managing addiction and other chronic diseases. To this end, the authors of this article performed a literature review of studies evaluating the use of ICTs in managing chronic diseases, including addiction. To determine whether these approaches really are effective and can make a difference in patient’s lives, the review focused on ICT-based interventions for which RCTs that assessed health outcomes had been conducted. The RCT methodology, in which study participants are randomly assigned to one of two or more treatment and control groups so that the basic characteristics of all groups can be considered equivalent, provides stronger evidence for addressing this question than other approaches, in which experimental groups may not be equivalent, potentially resulting in less solid conclusions (Campbell and Stanley 1963; Finney 2008). Studies that only assessed such issues as usability, usage, or acceptance of ICT-based interventions or in which the technology was used solely to collect data and not for intervention, as well as case studies and ethnographic and correlational analyses, were excluded from the review because although they can make very important contributions, they cannot answer questions regarding effectiveness.

To identify RCTs evaluating the effectiveness of ICTs in the management of chronic diseases, the investigators examined articles in the Web of Knowledge and PubMed databases, using three categories of search terms:^2^

- Technology-related terms, such as technology, computer, personal computer, mobile device, smartphone, cell phone;
- Medical terms, such as chronic disease, chronic illness; and
- Function-related terms, such as management, self-management, self-monitoring, self-educating, and patient education.

Prior to this broad search including all chronic diseases, a specific search for ICTs supporting recovery from addiction was conducted. This analysis identified six articles reporting on randomized trials, which are included in the analyses described below. Because of this relatively small number of studies, however, the search was broadened to include all chronic diseases.

The investigators initially identified hundreds of articles that were associated with these search terms. Further examination narrowed the list to 46 articles that reported on randomized trials of ICTs. Of those, 12 addressed brief interventions and therefore were not included in further analysis. (For more information on these studies, see the article by Cunningham and colleagues, pp. 320–326 in this issue). The remaining 34 studies are summarized in the table. Analyses of various characteristics of these studies yielded the following findings (for the specific references within each subgroup, see the table):

- The number of RCTs on use of different technologies for chronic disease management has increased substantially in recent years. Thus, the literature search identified 6 studies published prior to 2003, 9 studies published between 2003 and 2006, and 19 published between 2007 and 2010.
- Of 34 studies, 21 used personal computer–based approaches, 9 used land-line telephone-based approaches, 3 used mobile phone–based approaches, and 1 used a television-based approach. One study used both personal computers and mobile phones.
- ICTs have been evaluated for a range of chronic condition. Thus, 10 RCTs were targeted at AOD use; 1 addressed a combination of chronic diseases (heart disease, lung disease, and diabetes); 7 assessed management of diabetes; 3 addressed cancer; 4 focused on heart disease or heart failure; 5 addressed smoking; 2 were targeted at depression; and 1 each addressed chronic headache, HIV infection, high blood pressure, and chronic lung disease.

The RCTs analyzed used different intervention strategies, including monitoring, self-management, and push or pull technologies.^3^ Thus, 13 ICTs included monitoring, self-management, and push technology; 14 involved some form of stand-alone “therapy” (excluding screening and brief interventions); and 6 included monitoring and therapy but no push technology.

### Effectiveness

Overall, 29 of 34 interventions studied yielded positive effects on the outcomes measured, 1 study demonstrated a weak effect, 2 studies found a dose response, and only 2 studies yielded no effects. The combination of monitoring and push technology seemed to be particularly important for effectiveness. Thus, all 13 ICTs that used monitoring and push-based interventions found significant positive effects, whereas only 9 of 14 therapy interventions found positive effects. The six studies that included both monitoring and “therapy” but no push technology produced positive outcomes. Two interventions generated a dose-response effect but no intent-to-treat effects.^4^ One of the studies focusing on diabetes patients also addressed cost-effectiveness, suggesting a return on investment similar to that found with other interventions for this disorder.

With respect to the chronic disorders targeted, all 10 AOD studies yielded a positive effect, as did 6 of 8 studies among diabetic patients, all 5 studies on heart disease or heart failure, all 3 cancer studies, 3 of 5 smoking studies, and 1 of 2 depression studies.

## Discussion

Several other investigators previously have reviewed the use of computer-based systems in the management of chronic diseases. These reviews generally found encouraging results, albeit with a common caveat of methodological limitations. For example, in a review of 10 studies assessing mental health problems (e.g., obsessive compulsive disorder and panic), Barlow and colleagues (2005) reported positive results but noted that they were “mainly based on small samples, lacked long-term follow-up, and failed to address cost-effectiveness” (p. 272). Similarly, Murray and colleagues (2005) reviewed the Cochrane database and identified 24 randomized trials of ICT-based chronic disease interventions. The studies found generally positive effects on knowledge, social support, and behavioral and clinical outcomes of patients using these technologies compared with nonusers. However, the authors again suggested that future studies are needed to improve in quality and sample size to determine the best type of and best way to deliver ICT-based interventions and to establish the mechanisms through which ICT-based interventions affect different groups of people with chronic illness.

Despite the promising results, however, it is important that “policy makers should be cautious about recommending increased use and investment in unevaluated technologies” (Currell et al. 2000, p. 2). This is particularly true for smartphones, for which the proliferation of unevaluated applications is evident (Bewick et al. 2008). However, high-quality randomized trials of smartphone applications in the management of chronic diseases still are rare. Results of such studies are just now beginning to appear, and it is too early to generalize about their usefulness.

For other technologies, however, existing results offer encouragement and guidance for their use in chronic disease self-management, as indicated by the literature review presented here. The most dramatic effects appear to be seen with interventions using push technology that combines monitoring with tailored information, social support, and automated reminder systems that alert both the patient/family and the clinical team when a predetermined indicator surpasses a threshold.

For the next step, it is important that these evaluations move beyond assessing effects in the controlled setting of a clinical trial (i.e., beyond efficacy studies) to assessing effects in real-world settings (i.e., effectiveness trials) and comparative effectiveness analyses. The literature review presented here identified only one cost-effectiveness study of chronic disease self-management using ICTs. That study concluded that costs to produce one additional quality-of-life year were similar to those of other accepted interventions (Handley et al. 2008). However, more cost-effectiveness studies combined with randomized trials of larger sample sizes are needed to address this issue.

The studies reviewed here demonstrate that it is possible to complete RCTs of ICT-based interventions. However, other types of research are needed as well. For instance, these novel technologies typically are incorporated in an existing treatment system that may not be welcoming to them. For example, some residential substance use treatment programs do not allow cell phones or Internet access. Therefore, studies are needed to identify the key considerations and support systems that must be addressed to make implementation of ICT-based approaches a long-term success. Furthermore, ethnographic studies should be conducted to better understand the reasons why a given technology is successful or unsuccessful within different types of settings.

To date, few randomized trials of smartphone applications in the management of chronic diseases have been published. This is understandable given the relative recent, albeit rapid, appearance of these resources. Now that the technology has advanced and smartphone applications are widely available, however, it is important to pursue them and research their efficacy, because with such proliferation the risk of adopting ineffective and potentially harmful applications increases. At a recent National Institute on Drug Abuse (NIDA) Blending Conference, Miller (2010) reported on studies demonstrating wide variations in the quality of addiction counselors and the attendant risks. Similarly, some ICT (particularly smartphone) applications may be very helpful, whereas others may be dangerous (e.g., if they advocate unproven interventions or claim unproven effects). Federal agencies appear to be taking steps to address this issue by suggesting that the U.S. Food and Drug Administration (FDA) form a mental health regulatory commission (Thompson 2010). The NIH, along with other Federal agencies, are sponsoring a series of meetings (such as the November 2010 Health Summit organized by the Foundation for the National Institutes of Health [http://www.mhealthsummit.org], where current research technologies as well as future directions were addressed) that may lead to initiatives aimed at evaluating these technologies. Such initiatives have important implications for the research community.

One issue that affects the validation process for technological solutions is speed. Some studies identified in the literature review presented here are more than 10 years old. Thus, they can only provide important historical contributions in a field that is changing rapidly. For instance, a few years ago smartphones with the ability to conduct two-way video chats still were on the horizon—now they exist. In this rapidly changing environment, Federal grants support evaluation studies that take 5 years to complete. As a result, although these studies have value, some findings can be obsolete before the study is completed. Randomized trials are an important research tool, but in certain situations they need a shorter timeline to completion. This requires a new research paradigm. One solution may be to find ways to complete recruitment of participants in months rather than years. Another may be to use high-end technologies that, although they still are too expensive to be commonly used now, may be ubiquitous in a few years. One can, of course, argue that other addiction treatments are not held to such high standards of evidence as RCT evaluations, and therefore neither should technological solutions. However, just because other treatments have not undergone a scientific validation process of their benefits and costs (Popovici et al. 2008), that does not mean that this practice is ideal. There has been extensive support for the use of certain evidence-based practices in the treatment of addictions, and smartphone applications also should fall into this category.

It is important to note that the literature analysis presented here does not represent an exhaustive search and the conclusions reached are preliminary. For example, studies other than randomized trials likely also could offer important insights into the issues addressed here. In addition, other databases could have been searched. Nevertheless, the analysis allows two main conclusions. First, researchers are beginning to understand the requirements for producing high-quality interventions using smartphones and ICTs. Second, a much better evidence base (addressing effectiveness and costs) needs to be established and, because of the rapid evolution of the technology, needs to be updated continually and rapidly. This requirement calls for a new research paradigm. Thus, although investigations into the full potential of these interventions have begun, there still is a long way to go.

## Figures and Tables

**Table:** List of Studies Reviewed for This Analysis

**Author**	**Year**	**Disease**	**Effect/Results**	**Intervention**	**Technology**	**Other**
Adams et al.	2009	Diabetes	No effect	Therapy	Computer	
Alemi et al.	1996	Alcohol and other drugs (AODs)	Positive effect	Therapy	Telephone	
Bickel et al.	2008	AODs	Positive effect	Therapy	Computer	
Billipp	2001	Depression	Weak effect	Therapy	Computer	
Devineni and Blanchard	2005	Headache	Positive effect	Therapy	Computer	
Etter	2005	Smoking	No effect	Therapy	Computer	
Friedman et al.	1996	High blood pressure	Positive effect	Monitor-Therapy	Telephone	
Glasgow et al.	2002	Diabetes	Positive effect	Monitor-Push	Telephone	
Gustafson et al.	1999	HIV	Positive effect	Monitor-Therapy	Computer	
Gustafson et al.	2001	Cancer	Positive effect	Monitor-Push	Computer	
Gustafson et al.	2008	Cancer	Positive effect	Monitor-Therapy	Computer	
Handley et al.	2008	Diabetes	Positive effect	Monitor-Therapy	Telephone	
Harris et al.	2009	AODs	Positive effect	Monitor-Push	Computer	
Hester et al.	2009	AODs	Positive effect	Therapy	Computer	
Japuntich et al.	2006	Smoking	Dose response	Therapy	Computer	
Kay-Lambkin et al.	2009	AODs, depression	Positive effect	Therapy	Computer	
Kramer et al.	2009	AODs	Positive effect	Therapy	Television	
Lindsay et al.	2008	Heart disease	Positive effect	Therapy	Computer	
Lorig et al.	2006	Heart disease, lung disease, diabetes	Positive effect	Monitor-Push	Computer	
McKay et al.	2005	AODs	Positive effect	Monitor-Push	Telephone	
McKay et al.	2010	AODs	Positive effect	Monitor-Therapy	Telephone	
Noh et al.	2010	Diabetes	Positive effect	Monitor-Push	Cell phone, computer	
Patten et al.	2006	Smoking	Dose response	Therapy	Computer	
Riper et al.	2008	AODs	Positive effect	Monitor-Push	Computer	Telemedicine
Riper et al.	2009	AODs	Positive effect	Therapy	Computer	
Rodgers et al.	2005	Smoking	Positive effect	Therapy	Cell phone	Text message
Ruland et al.	2010	Cancer	Positive effect	Monitor-Push	Computer	
Scherr et al.	2009	Heart failure	Positive effect	Monitor-Push	Cell phone	
Schillinger et al.	2008	Diabetes	Positive effect	Monitor-Push	Telephone	
Shea and Ideatel Consortium	2007	Diabetes	Positive effect	Monitor-Push	Telephone	
Strecher et al.	2005	Smoking	Positive effect	Tailoring	Computer	
Stromberg et al.	2006	Heart failure	Positive effect	Monitor-Push	Computer	
Williams et al.	2007	Diabetes	Positive effect	Monitor-Therapy	Computer	
Woodend et al.	2008	Heart disease	Positive effect	Monitor-Push	Telephone	

## Footnotes

1 Mediation analyses attempt to identify the factors that are responsible for the effect of a variable on a given outcome. For example, if a smartphone-based intervention improves patient outcome by reminding the patient to take his or her medications regularly at the right time, then the reminder function would be considered the mediator of effectiveness. Conversely, a moderator analysis attempts to determine the outside factors that influence the intervention’s effect on outcome. For example, if in the example above the intervention was more effective in men than in women, then gender would be considered a moderator.

2 Other search terms that were not explicitly used but frequently encountered included (1) technology-related terms such as Internet, health information technology, telephone, telephony, multimedia, social media, patient health records, electronic health records, electronic medical records, and electronic practice management; (2) medical terms, such as cancer, heart disease, diabetes, stroke, obesity, arthritis, kidney disease, pulmonary, asthma, multiple sclerosis, chronic conditions, rheumatology, alcohol, drug abuse, and substance abuse; and (3) function-related terms, such as self-education, consumer informatics, patient decision aids, brief intervention, therapy, counseling, and data collection.

3 With push technologies, the computer initiates an action request to which the patient has to respond; in contrast, with pull technologies the user (i.e., patient) initiates the action or request.

4 An intent-to-treat analysis includes all participants recruited into a study, regardless of whether and to what extent they then actually participate in the intervention. Thus, a lack of an intent-to-treat effect indicates that the participants recruited into the experimental group when analyzed together did not show better outcomes than the control group. A dose-response analysis evaluates whether among those people who actually participate in the intervention those that participate to a greater extent show greater effects than those who participate to a lesser extent. Thus, the dose-response effect seen in the two studies discussed here indicates that participants who used the ICTs more extensively had better outcomes than those who used them less extensively.
